# Application of Image Fusion Algorithm Combined with Visual Saliency in Target Extraction of Reflective Tomography Lidar Image

**DOI:** 10.1155/2022/8247344

**Published:** 2022-02-27

**Authors:** Xinyuan Zhang, Yihua Hu, Shilong Xu, Fei Han, Yicheng Wang

**Affiliations:** ^1^State Key Laboratory of Pulsed Power Laser Technology, National University of Defense Technology, Hefei 230037, China; ^2^Anhui Province Key Laboratory of Electronic Restriction, National University of Defense Technology, Hefei 230037, China

## Abstract

Reflective tomography Lidar has been proved to be a new Lidar system with long distance and high resolution. The reflective tomography Lidar image is prone to clutter and artifacts; thus, it is important for space target recognition to extract the target from the image. In this study, we proposed image fusion algorithm combined with visual saliency could be applied to the target extraction of reflective tomography Lidar image, which can not only preserve the target information but also eliminate the clutter and artifacts in the image. The efficiency of this algorithm is shown by simulation and the experiment of the reflective tomography Lidar system. Also, we analyzed the main source of reflective tomography Lidar image artifacts and the reason why this algorithm could remove clutter and artifacts.

## 1. Introduction

Aerial and space target detection and identification have gained wide attention with the rapid development of aerospace science and technology. Reflective tomography Lidar has been proved to be a new Lidar system with long distance and high resolution [1], especially which is appropriate for the detection of targets in the dark background.

Developed from computed tomography (CT), the concept of laser reflective tomography (LRT) was firstly introduced by Parker et al. [2] working in Lincoln Laboratory in the United States in 1988. Knight et al. [3] further improved the Lidar experimental system by using streak cameras to increase the bandwidth of the detection circuit so as to improve the imaging resolution. In addition, abundant indoor imaging experiments have been carried out to verify the application prospect of LRT [4–6]. On this basis, Maston et al. [7] conducted a deep study of the theory of LRT and applied it to the imaging detection of space targets, such as satellites [8–10]; they also obtained reconstruction images of the satellite from a ground base Lidar system [11, 12]. In 2010, Murray et al. [13] from Areté company combined the reflective tomography Lidar with range compressed and realized the imaging accuracy of 0.15 m for the non-cooperative target with a size of 1m at a distance of 22.4 km, which further improved the imaging resolution of the reflective tomography Lidar.

It is important for space target recognition to extract the target from reflective tomography Lidar image on account of the process of image reconstruction and is easy to produce clutter and artifacts. The traditional target extraction algorithm is the threshold segmentation; it utilized the difference between the gray value of the target and the background from the image. Also, it classifies the pixels by setting the threshold value so as to realize the separation of the target and the background [14, 15]. This algorithm is not complex in principle and is easy to operate, but the segmentation effect mainly depends on the selection of segmentation threshold, and it can easily fail to produce moderate segmentation either insufficient or excessive. Iterative threshold algorithm is the most commonly used image automatic segmentation algorithm for the reflective tomography Lidar image at present. This algorithm is based on the closing thought; the advantage is that the amount of computation is not large; under certain conditions, the change of image grayscale is little affected, and some real-time image processing systems have been widely used [16]. However, the disadvantage of this algorithm is that it only uses the gray information of image pixels without considering the spatial correlation information between pixels, so the anti-interference effect is poor and satisfactory segmentation effect cannot be obtained.

Therefore, an algorithm that can automatically extract the target from reflective tomography Lidar image is necessary, and this algorithm is able to eliminate the clutter and artifacts while preserving the target information as much as possible. Basically, image fusion algorithm is focused to combine two or more images into a new image [17]; the fusion result can utilize the correlation of multiple images in space-time and make the image obtained after fusion have a more comprehensive and clear description of the scene [18, 19], all of these processes above aim to improve the utilization of image information and be more conducive to target detection. In this study, we proposed an image fusion algorithm combined with visual saliency, could use visual saliency detection to locate the target area, filter the effects of clutter and artifacts in the image, and generate the saliency map. Finally, the target image could be obtained by fusing the input image with the mean filtering of the saliency value of the 2D distribution.

The rest of this paper is organized as follows. In Section 2, we briefly review the principle of LRT. The whole process of image fusion algorithm combined with visual saliency was described in detail in Section 3. In Section 4, we built the system and verify the effectiveness of this algorithm through experiments. In Section 5, the main source of image artifacts and the reason why this algorithm could remove clutter and artifacts were analyzed by combination with the reflective tomography Lidar image. Finally, we draw a conclusion in Section 6.

## 2. Review of LRT

The structure inversion of the target is detected by LRT on obtaining the structure characteristic information of the target from the multiangle reflective wave. The basic principles of this method are to illuminate the object of laser at multiple angles, collect the reflective wave of the target at multiple angles, and reconstruct the 2D cross-sectional image of the target according to the reflectance projection distribution [20].

**Figure 1:**
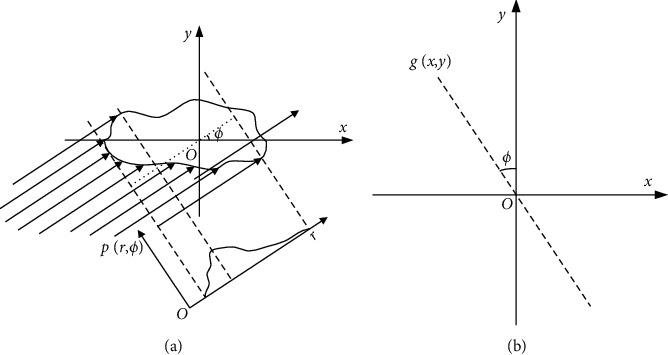
Schematic of the principle of LRT. (a) Target projection. (b) Data backprojection.

As shown in Figure 1(a), the parallel laser beams irradiate a 2D target, when the irradiation angle is *ϕ*. The reflectance projection distribution of the target at angle *ϕ* is defined as

(1) $$\begin{array}{c} p\left(r,\phi \right)=\int_{L_{r\mathrm{,\phi}}}f\left(x,y\right)\text{ds}, \end{array}$$

where *L*_*r*,*ϕ*_ is a straight line perpendicular to the direction of light, with a function of *r*=*x*  cos*ϕ*+*y*  sin  *ϕ*, and *f*(*x*, *y*) is the reflectance distribution of the target.

An angle projection image is shown in Figure 1(b); the parallel lines with arrows unfold in the laser beams' irradiate region, and the distance of the projection depends on the depth of the region irradiated by the laser. It should be noted that the reflectance projection distribution is actually nonzero only on the surface, as laser beams cannot penetrate the target. That is,

(2) $$\begin{array}{c} f\left(x,y\right)=0,\left(x,y\right)\notin D, \end{array}$$

where *D* is the set of points of the target surface. Therefore, the actual projection can be represented as

(3) $$\begin{array}{c} p\left(r,\phi \right)=\int_{L_{r,\phi }\cap D}f\left(x,y\right)\text{ds}. \end{array}$$

According to (3), we further deduced that the actual projection of the 3D target can be expressed as

(4) $$\begin{array}{c} p\left(r,\phi \right)=\iint_{S_{r,\phi }\cap B}f\left(x,y,z\right)\text{ds}, \end{array}$$

where *S*_*r*,*ϕ*_ is a plane perpendicular to the direction of light, *f*(*x*, *y*, *z*) is the reflectance distribution of the target, and B is the set of points of the 3D target surface.

## 3. Target Detection Method

### 3.1. Overview of General Process

Because the reflective tomography Lidar image is prone to clutter and artifacts, it is important to extract the target from the image, especially the artifacts reduction. Artifacts is the abnormal change of the gray level in the image which is not consistent with the target structure due to the imperfect measurement data or the error projection data judgment caused by various physical factor. Filtered backprojection (FBP) algorithm is the universal method for LRT to reconstruct the 2D cross-sectional image of the target, especially complete angle reconstruction [21]. Therefore, artifacts affect not only local areas but also the entire image. For example, a thin metal wire creates a stripe artifacts that covers itself and a large area around it.

**Figure 2:**
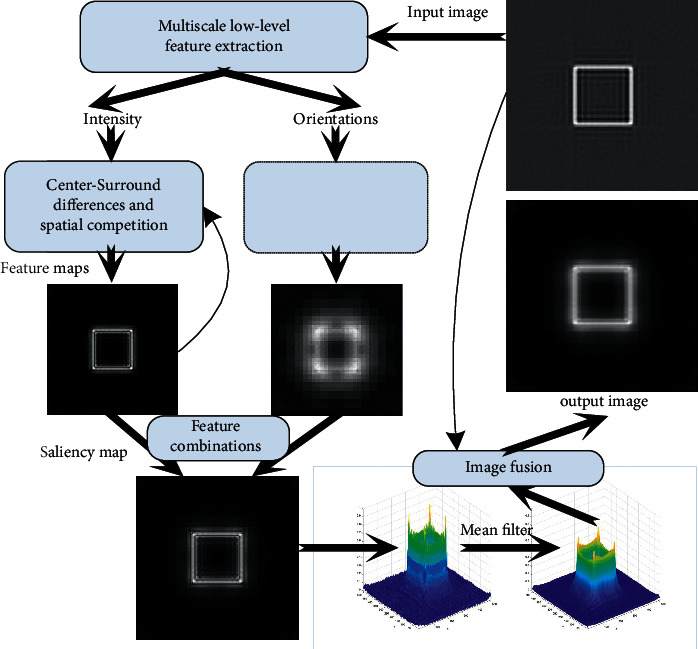
Flowchart of image fusion algorithm combined with visual saliency.

Insufficient or excessive segmentation is likely to be generated by the traditional segmentation algorithm for the artifacts in the image [22]. To this end, we proposed an image fusion algorithm combined with visual saliency and applied it to the target extraction of reflective tomography Lidar image; flowchart of this algorithm is shown in Figure 2. Firstly, this algorithm uses visual saliency detection to locate the target area, filters the effects of clutter and artifacts in the image, and finally generates the saliency map. Specifically, inputting the reconstructed image by FBP and the multiscale low-level feature extraction are carried out to obtain the intensity and orientations features at the first step; then, the center-surround differences and spatial competition are used to get the feature maps and the feature combinations get the saliency map. Afterwards, the mean filter is applied to the salient values of the 2D distribution to make the image smooth. Finally, the target image is obtained by fusing the input image with the mean filtering of the saliency value of the 2D distribution.

### 3.2. Visual Saliency Detection

Visual saliency detection refers to the extraction of salient regions in images, that is, areas of interest to human beings, by simulating human visual characteristics through intelligent algorithms. The visual attention model based on saliency was proposed by ITTI in early 1998 [23] and further improved in Nature in 2001 [24], which is a classic bottom-up saliency detection model based on bottom salient feature calculation. Its realization process is roughly divided into Gaussian filtering, calculation of the bottom space feature map and calculation of the salient map.

Based on the ITTI model, we formed the general process of visual saliency detection of LRT images. Firstly, Gaussian pyramid of image intensity and orientations was constructed by the Gaussian sampling method, and then, the intensity and orientation feature maps were calculated by Gaussian pyramid. Finally, the intensity and orientation saliency maps were obtained by combining the feature maps of different scales, and the final visual saliency map was obtained by adding them together. This algorithm does not require training and learning process, but can complete saliency map calculation by pure mathematical methods. Comparing with the spatial frequency content (SFC) model proposed by Reinagel et al. on target detection, and the experimental results showed that the ITTI model has sound robustness to noise, while the SFC model does not. It should be pointed out that the input of the algorithm in this paper is a static grayscale image with the size of 2048×2048, and we will describe the detailed process of each step in the following sections.

The construction of Gaussian pyramid includes intensity and orientations. Intensity is to do Gaussian downsampling on the grayscale image so as to obtain grayscale images under nine scales to construct intensity Gaussian pyramid; then, the Gabor filter is used to construct Gabor direction pyramid. After obtaining the intensity and orientation Gaussian pyramids mentioned above, the Center-Surround method is used to calculate the corresponding feature images, in which Center (c) refers to fine scale and Surround (s) refers to the coarse scale; the calculation method is as follows:

(5) $$\begin{array}{c} I\left(c,s\right)=I\left(c\right)⊖I\left(s\right),\\ O\left(c,s,\theta \right)=\left|O\left(c,\theta \right)⊖O\left(s,\theta \right)\right|. \end{array}$$

While, *c* ∈ {2,3,4}, *θ* ∈ 0°, 45°, 90°, 135°, and *s*=*c*+*δ*. The ⊖ operation in the formula means that matrix subtraction is performed after adjusting the size of the two images to the same size. *I* represents 6 intensity feature maps, and *O* represents 24 orientation feature maps, so a total of 30 feature maps are generated. Then, the following formula is used to calculate the intensity saliency map and orientation saliency map, respectively:

(6) $$\begin{array}{c} \bar{I}=\overset{4}{\underset{c\mathrm{=2}}{⊕}}\overset{c\mathrm{+4}}{\underset{s=c\mathrm{+3}}{⊕}}N\left(I\left(c,s\right)\right),\\ \bar{O}=\underset{\theta \in 0^{\circ },45^{\circ },90^{\circ },135^{\circ }}{\sum }N\left(\overset{4}{\underset{c\mathrm{=2}}{⊕}}\overset{c\mathrm{+4}}{\underset{s=c\mathrm{+3}}{⊕}}N\left(O\left(c,s,\theta \right)\right)\right). \end{array}$$

The ⊕ operation in the formula means that matrix addition performs after adjusting the size of the two images to the same size. Then, the intensity and orientation saliency maps are obtained, and the final saliency map are obtained by

(7) $$\begin{array}{c} S=\frac{1}{2}\left(N\left(\bar{I}\right)+N\left(\bar{O}\right)\right). \end{array}$$

In fact, salient targets are often detected according to the set threshold in target detection. As the set threshold gradually decreases, the salient targets obtained gradually increase and the detection time also increases. However, the saliency map has the problem of blurring the boundary, so it is not an ideal way to extract the target by using the saliency map to segment the reflective tomography.

### 3.3. Image Fusion

As mentioned above, using the saliency map is not the best way to extract the target and to segment the reflective tomography Lidar image due to the problem of blurring the boundary of the salient map. Therefore, it is necessary to propose an image fusion algorithm that can fuse the salient map with an input image to obtain a new target image. The image fusion algorithm requires two or more images to be fused and should have been registered well, and the pixel bit width is the same; otherwise, the fusion effect is not expected [25, 26].

The saliency value of a region in the saliency map can actually describe the degree of interest, that is to say, the saliency value of 2D distribution can actually be considered as the weight of each pixel of the input image. Also, the fusion image obtained by directly multiplying the weight with the input image can be used as a new segmented image. However, the saliency value of the 2D distribution has a large number of active peaks; thus, the disadvantage of this algorithm is that the target area near the active peaks in the image will be enhanced, while its surrounding area will be suppressed; to solve this problem, we need to smooth the saliency value data of 2D distribution. Mean filtering is a typical linear filtering algorithm [27]; it means giving a template to the target pixel on the image, which includes the neighbouring pixels around it, and then replaces the original pixel value with the average value of all the pixels in the template. The calculation formula is as follows:

(8) $$\begin{array}{c} g\left(x,y\right)=\overset{i=x+m}{\underset{i=\mathrm{x-m}}{\sum }}\overset{j=y+m}{\underset{j=y+m}{\sum }}\frac{f\left(x,y\right)}{{\left(2m\mathrm{+1}\right)}^{2}}. \end{array}$$

While *m* is the window size and (2*m*+1)^2^ is the total number of pixels in the template including the current pixel, and it can be fused with the input image using the following formula:

(9) $$\begin{array}{c} IR{}^{\prime }\left(i,j\right)=g\left(i,j\right)\times IR\left(i,j\right). \end{array}$$

Furthermore, in order to remove the influence of the noise base, we normalize the segmented image to a unified range and then restore it to the grayscale range, as shown in the following formula:

(10) $$\begin{array}{c} IR=255\times \frac{IR\left(i,j\right)-\min\left[IR\left(i,j\right)\right]}{\max\left[IR\left(i,j\right)\right]-\min\left[IR\left(i,j\right)\right]}. \end{array}$$

## 4. Experiment and Result

**Figure 3:**
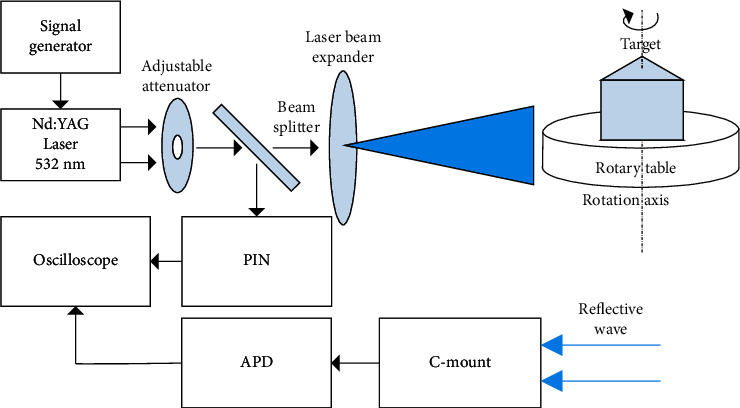
Experimental system of LRT.

An experimental system for LRT is constructed, and the experimental setting is shown in Figure 3. The laser beam passes through the beam splitter and then goes through the adjustable attenuator and the beam expander to enlarge the emitting laser beam so that the beam at the target can completely cover the target surface within a certain distance. At the signal receiving end, a C-mount industrial lens is used as the receiving device for reflecting laser pulse signals, and the detector adopts a high-sensitivity Si-based avalanche photodiode (APD) single pixel detector to directly receive the reflective wave. In addition, a reference signal is added and received by a PIN diode, which is used to measure the laser pulse amplitude to correct the reflective wave amplitude.

The experimental system uses a 532 nm Nd:YAG laser with a pulse width of 1 ns, a detection circuit bandwidth of 1.5 GHz, and an oscilloscope sampling rate of 10 GSPs. The target is a triprism model with a height of 1.0 m and a base length of 0.8 m, 1.0 m, and 1.0 m. The three sides were sprayed with green paint, black paint, and aluminum foil, respectively, and used different reflectivity materials. The stepping angle of the motor is set as 1 degree to carry out full angle detection around the target in 360 degrees. A total of 360 groups of reflective wave data of laser reflective from different angles of the target are collected, and the distance between the signal receiving device and the rotation center of the target is 39.7 m.

**Figure 4:**
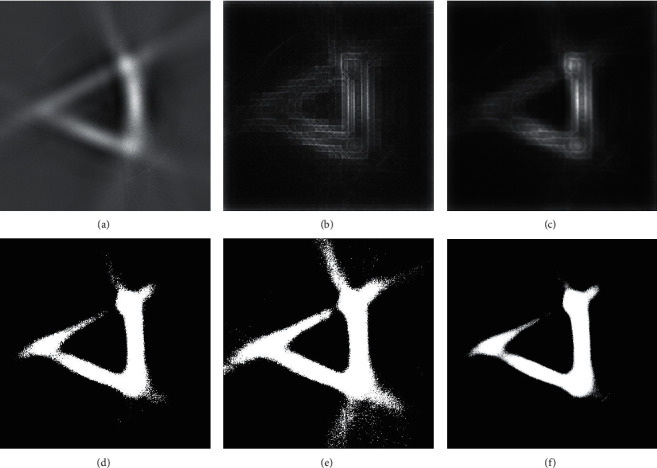
Target image. (a) Reconstructed image by FBP. (b) Saliency map. (c) The image after the processing of the algorithm; threshold segmentation image. (d) High-threshold image. (e) Low-threshold image. (f) Obtained by iterative threshold algorithm.

After the reflective waveform is registered by the reference screen method, the 2D cross-sectional image of the target reconstructed by FBP, and this is shown in Figure 4(a). It can be seen that there are clear triangular shapes, and the brightness contrast of aluminum foil facade is much greater than those of green paint facade and black paint facade. However, this image has serious artifacts; the contour is extreme fuzzy and exists high clutter base. The saliency map obtained after saliency detection of the input image is shown in Figure 4(b), and it is clear that the artifacts in the image are significantly reduced, but the edges become more blurred.

**Figure 5:**
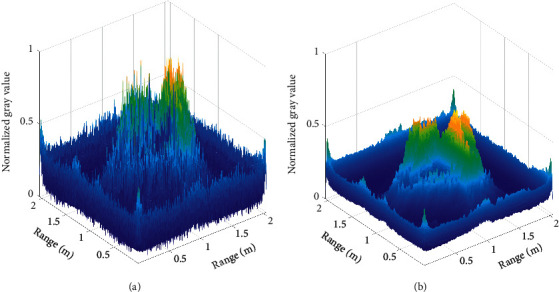
(a) 3D display of the salient map. (b) 3D display of the salient map after mean filtering.

Figure 5(a) is the 3D display of this saliency map, and Figure 5(b) is the 3D display of the salient map after mean filtering. The image after the processing of the algorithm, shown in Figure 4(c), can be obtained by fusing with the input image. The threshold segmentation images obtained by the typical threshold value show that the image obtained by the high threshold value, as shown in Figure 4(d), loses various target information, and the image obtained by the low threshold value is shown in Figure 4(e). Although the target information is retained, a large number of clutters and artifacts are also retained.

The threshold segmentation image obtained by iterative threshold algorithm is shown in Figure 4(f), which is the most common method that used image automatic threshold segmentation algorithm for the reflective tomography Lidar image at present. Compared with the above threshold segmentation image, the image after the processing of the algorithm is shown in Figure 4(c); it can be found that the threshold segmentation image which is shown in Figure 4(f) of the black paint facade is lacking; a large number of artifacts emerge in the corresponding edge information and the corresponding edges on both sides of the aluminum foil facade, and the image processed by the algorithm in this paper retains complete black paint facade on the edge of the corresponding information. At the same time, the artifacts are effectively eliminated. From the experimental results, comparing Figure 4(c) with Figures 4(d)–4(f), it can be found that the proposed algorithm not only retains the target information but also removes clutter and artifacts better, and the problem mentioned in the study that “traditional image artifacts' segmentation algorithms are prone to cause insufficient or excessive segmentation“ can be successfully solved by the proposed algorithm.

## 5. Combination with the Reflective Tomography Lidar Image

Furthermore, in order to verify the effectiveness of this algorithm, the reflective waveform simulation system of the reflective tomography Lidar is established, which is combined with reflective tomography Lidar. 3DS Max is used to generate a 1 : 1 3D model of the detection target aircraft, in which the wingspan of the aircraft is about 13 meters, the length is about 19 meters, and the fuselage height is about 2.8 meters. The distance between the Lidar and the target coordinated origin is set at about 10 km, the elevation angle of the laser beam center relative to the target scene is set at 0 degrees, and the divergence angle of the laser beam is set at 1 mrad.

**Figure 6:**
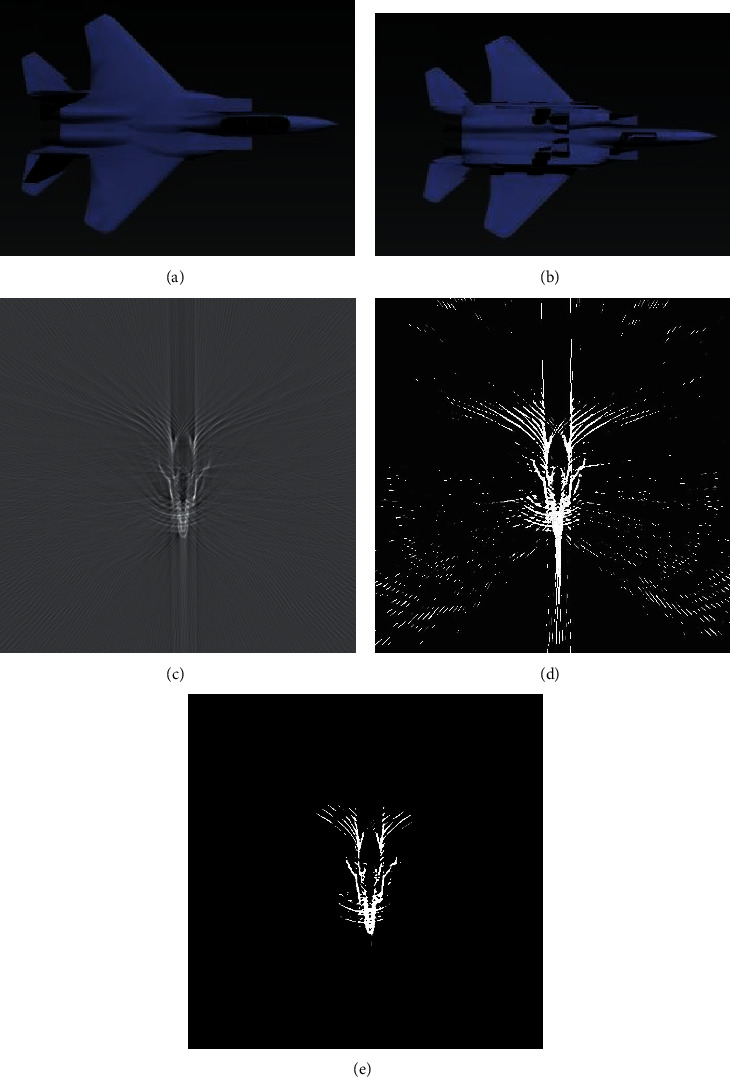
Target prototype drawn by 3DS Max. (a) Vertical view. (b) Upward view. (c) Reconstruction image by FBP; threshold segmentation image. (d) Low-threshold images. (e) High-threshold images.

**Figure 7:**
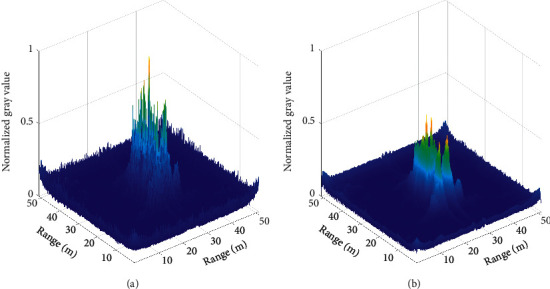
(a) 3D display of the salient map. (b) 3D display of the salient map after mean filtering.

As shown in Figure 6, the reconstructed image by FBP has clutter and artifacts, and the low threshold image obtained by the traditional threshold segmentation algorithm retains numerous artifacts, while the high threshold image loses abundant target information. Furthermore, we carry out mean filtering for the saliency value of 2D distribution, as shown in Figure 7. It can be seen that the previous 3D display of the salient map after mean filtering is much smoother than the 3D display of the salient map, and the activity peaks in the image are significantly suppressed.

**Figure 8:**
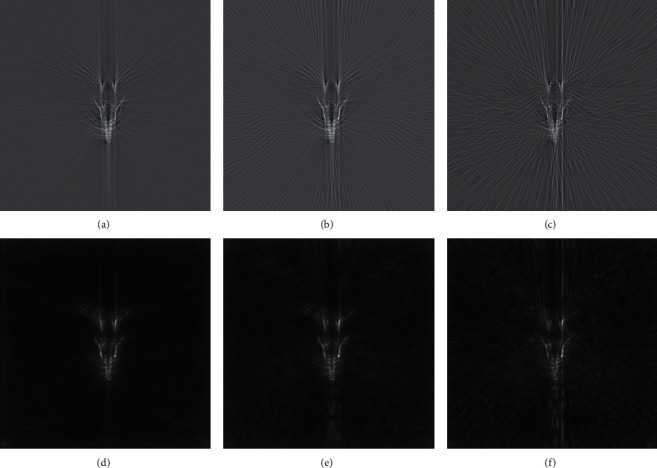
Reflective tomography Lidar image: the sampling interval is (a) 1 degree; (b) 2 degrees; (c) 4 degrees. Fusion image: the sampling interval is (d) 1 degree; (e) 2 degrees; (f) 4 degrees.

Reflective tomography Lidar images at different sampling intervals are shown Figure 8; it can be seen that, as the sampling interval becomes larger, the target identification in the image becomes worse, and numbers of artifacts become more. However, the segmented images processed by the algorithm are shown in Figure 8(d)–8(f), and the clutter and artifacts are obviously removed.

Finally, the reasons why this algorithm can eliminate clutter and artifacts are analyzed as follows. The Gaussian sampling method is used in the image intensity of Gaussian pyramid structure; each layer of the pyramid is composed of a layer of the pyramid half downsampling, so the image of the clutter in the process of downsampling is affected by average area around such weakening; as a result, this algorithm can effectively filter clutter. The causes of artifacts in the reflective tomography Lidar are more complex and need to be analyzed by reference to CT and photo acoustic tomography (PAT). We believe that the star fringe near the structure is due to the undersampling of the projection [28], that is to say, the limited projection data will cause the computer to small targets with sharp edges and relevant information from the registration error, including small stripe seems to emanate from the edge of the dense structure, as the simulation and experimental results are given. Since the reflective waveform is registered by the reference screen method, the motion artifacts [29] can be ignored. At the same time, considering the difference in relative motion and detection model between reflective tomography Lidar and CT, the spiral artifacts [30] and cone-beam artifacts [31] are completely absent. In addition, there is no significant difference among the reflectivity of the detected target surface, so the partial volume effect artifacts [32] can be ignored. In summary, the undersampling artifacts are the main source of artifacts in the reflective tomography Lidar image, and the main reason why the algorithm could remove the artifacts is that four orientations parameters are selected by the Gabor filter in the process of Gaussian sampling to generate orientation features for filtering, so as to make the positioning of feature points more accurate.

## 6. Conclusion

In conclusion, we proposed an image fusion algorithm combined with visual saliency and applied it to the target extraction of the reflective tomography Lidar image. Verification was proceeded in the simulation model and the experimental system, and this algorithm is shown to work well in extracting the target from the image. Compared with the traditional threshold segmentation algorithm, this algorithm could not only preserve the target information but also eliminate the clutter and artifacts in the image. Besides, the 3D model of the aircraft was detected by reflective tomography Lidar, and this algorithm has been proved to be able to work effectively in eliminating the star artifacts of the aerial target in the reflective tomography Lidar images at different sampling intervals.

Furthermore, there would be a natural rationality to expand and apply the algorithm to the detection and recognition of space target. It should be pointed out that the current experimental system has the problem of insufficient detection range; however, the detection range requires reaching at least 50 km in real space environment. With this algorithm, reflective tomography Lidar could be developed into an integrated detection and identification for aerial target or space target approach. Detection of complex space targets with inhomogeneous reflectivity distribution and experiments on further range can be focused in future works.

## Data Availability

The data used to support the findings of this study are available from the corresponding author upon request.
